# Diagnosis-linked antibiotic prescribing in Swedish primary care - a comparison between in-hours and out-of-hours

**DOI:** 10.1186/s12879-020-05334-7

**Published:** 2020-08-20

**Authors:** Olof Cronberg, Mia Tyrstrup, Kim Ekblom, Katarina Hedin

**Affiliations:** 1 Växjöhälsan Primary Healthcare Center, VC Växjöhälsan, Hjortvägen 1, 352 45 Växjö, Sweden; 2 Department of Research and Development, Region Kronoberg, Växjö, Sweden; 3 Department of Clinical Sciences in Malmö, Family Medicine, Lund University, Malmö, Sweden; 4 Lundbergsgatan Primary Health Care Centre, Malmö, Sweden; 5 Department of Medical Biosciences, Clinical Chemistry, Umeå University, Umeå, Sweden; 6 Futurum, Region Jönköping County and Department of Health, Medicine and Caring Sciences, Linköping University, Linköping, Sweden

**Keywords:** Antibiotic prescribing, Diagnosis-linked prescription, Electronic health records, Infectious disease, In-hours, Out-of-hours service, Primary care

## Abstract

**Background:**

The rise in antibiotic resistance is a global public health concern, and antibiotic overuse needs to be reduced. Earlier studies of out-of-hours care have indicated that antibiotic prescribing is less appropriate than that of in-hours care. However, no study has compared the out-of-hours treatment of infections to in-hours treatment within the same population.

**Methods:**

This retrospective, descriptive study was based on data retrieved from the Kronoberg Infection Database in Primary Care (KIDPC), which consists of all visits to primary care with an infection diagnosis or prescription of antibiotics during 2006–2014. The purpose was to study the trends in antibiotic prescribing and to compare consultations and prescriptions between in-hours and out-of-hours.

**Results:**

The visit rate for all infections was 434 visits per 1000 inhabitants per year. The visit rate was stable during the study period, but the antibiotic prescribing rate decreased from 266 prescriptions per 1000 inhabitants in 2006 to 194 prescriptions in 2014 (mean annual change − 8.5 [95% CI − 11.9 to − 5.2]). For the out-of-hours visits (12% of the total visits), a similar reduction in antibiotic prescribing was seen. The decrease was most apparent among children and in respiratory tract infections.

When antibiotic prescribing during out-of-hours was compared to in-hours, the unadjusted relative risk of antibiotic prescribing was 1.37 (95% CI 1.36 to 1.38), but when adjusted for age, sex, and diagnosis, the relative risk of antibiotic prescribing was 1.09 (95% CI 1.08 to 1.10). The reduction after adjustment was largely explained by a higher visit rate during out-of-hours for infections requiring antibiotics (acute otitis media, pharyngotonsillitis, and lower urinary tract infection). The choices of antibiotics used for common diagnoses were similar.

**Conclusions:**

Although the infection visit rate was unchanged over the study period, there was a significant reduction in antibiotic prescribing, especially to children and for respiratory tract infections. The higher antibiotic prescribing rate during out-of-hours was small when adjusted for age, sex, and diagnosis. No excess prescription of broad-spectrum antibiotics was seen. Therefore, interventions selectively aiming at out-of-hours centres seem to be unmotivated in a low-prescribing context.

## Background

The rise of antibiotic resistance is a global public health threat according to the World Health Organization [1], and antibiotic overuse is common and results in medicalization, unnecessary costs, and increased antibiotic resistance [2]. However, studies on antibiotic prescribing in primary care regardless of indication show a high level of variability between physicians in different countries [3–5].

In primary care in-hours (IH) are usually office hours (in Sweden 08:00 to 17:00) during business days, and out-of-hours (OOH) are the remaining hours. Earlier studies of OOH care have suggested that compared to IH care there are lower adherence to antibiotic guidelines [6, 7], a higher antibiotic prescribing rate [8, 9], a higher rate of prescriptions for broad-spectrum antibiotics [8], and more antibiotic prescriptions during weekends than weekday evenings [10]. In a qualitative study from Belgium, the physicians reported that the threshold for prescribing antibiotics was lower during OOH, but the choice of antibiotics was the same [11]. A more recent Belgian OOH study showed a high antibiotic prescribing rate for all indications, a high rate of not using recommended antibiotics, and an overuse of quinolones [12]. However, a Dutch study found the prescribing quality to be appropriate, and the higher rates of prescribing in OOH were explained by a different population of presenting patients [13]. No previous study has compared the OOH treatment of infections to IH within the same population.

Although Sweden belongs to the European countries with low levels of antibiotic prescriptions, there is still room for improvement [14]. Previous registry-based studies in Sweden have shown a significant reduction in antibiotic prescriptions over the last decade, but these studies have not included OOH [15–17]. Several Swedish national guidelines concerning the evaluation and treatment of infectious diseases have been published [18–22], and generally these guidelines aim at better diagnostics, fewer antibiotics, and more targeted treatments.

Because visits for infectious diseases are common at OOH centres, it is important to evaluate whether OOH visits are associated with increased antibiotic prescribing rates because this would warrant interventions in OOH settings.

The purpose of the study was to describe the trends in antibiotic prescribing over time and to compare diagnosis-linked prescribing in general and in detail between IH and OOH in the same population.

## Methods

### Description of the study population

In 2014, Kronoberg County in southern Sweden had 189,128 inhabitants, which was equal to 2% of the Swedish population [23]. During 2014, there were a total of 243,502 physician visits for all causes and 238,164 other visits (nurses, physiotherapists, behavioural therapists) in primary care, thus there were 1300 physician visits and 1300 other visits per 1000 inhabitants.

During the study period, the number of primary healthcare centres (PHCCs) varied between 28 and 35, with 1–8 family physicians each. There were approximately 100 family physician positions and 50 junior physician positions. At the study start, all PHCCs were publicly run, but since March 2009 a third of the PHCCs have been privately run due to new legislation allowing publicly funded private PHCCs.

At the PHCCs, the patient normally booked an appointment through a telephone call with an office nurse who assessed if the patient needed a physician visit. IH were business days 08:00 to 17:00. In the region there were two OOH centres (OOHCs), and the PHCCs staffed the OOHCs with physicians. Patients were supposed to call a nurse triage first, but could also walk in. The visit fees were the same as for IH visits. Home visits were rare, and usually only performed for urgent cases at elderly care homes. Nurses at the OOHCs were responsible for phone advice, and there was also a national phone advice number for patients where nurses provided medical advice. At the time of the study, no Internet services were available.

OOHC 1 served approximately 125,000 inhabitants and was situated in the neighbourhood of the hospital in city 1. During 2006–2007 the centre was open from 17:00 to 24:00 on weekdays and from 08:00 to 24:00 on weekends and holidays. From 2008 the centre closed at 21:00. Walk-in patients met a nurse who assessed whether a meeting with a physician was warranted.

OOHC 2 served approximately 63,000 inhabitants and was situated at the emergency department of the hospital in city 2. During 2006–2007 the centre was open from 17:00 to 08:00 on weekdays and around the clock on weekends and holidays. From 2008 the centre closed at 21:00. Walk-in patients generally got to see a physician.

### The Kronoberg infection database in primary care (KIDPC)

This retrospective, descriptive study was based on data from the KIDPC database, which contains information on all visits with an infection diagnosis and all antibiotic prescriptions with or without a visit in primary care in Kronoberg County in 2006–2014. Annually, there were on average 86,000 visits for infections and 43,000 antibiotic prescriptions reported in the database.

The data in the KIDPC were extracted from the electronic medical records (EMR) used in Kronoberg County (Cambio Cosmic software, Cambio Healthcare Systems AB, Linköping, Sweden) at one instance in 2015 using BusinessObjects (SAP AG, Walldorf, Germany). These data contain detailed information about the patients (age, sex, anonymous ID), the visits (PHCC, geography, IH or OOH), the care providers (physicians, nurses), the investigations (diagnostic tests, x-rays, cultures), and the prescriptions (drugs, dosages, durations). The data were linked together using the anonymous patient ID and visit date. For all physician visits, at least one diagnosis was registered according to the simplified Swedish primary care edition of the *International Classification of Disease and Related Health Problems – Tenth Revision* (ICD-10) [24]. The diagnoses were validated and grouped into four main groups and several subgroups by one of the authors (OC) according to recommendations by Public Health Agency of Sweden [25]. The main groups are respiratory tract infections (RTIs), urinary tract infections (UTIs), skin and soft tissue infections (SSIs), and other infections. The RTI group includes ear infections, and the UTI group includes urogenital infections. The other infections group includes eye infections, gastrointestinal infections, and rare infections (See Additional file 1). Because at least one diagnosis had to be recorded for each physician visit, the data set is considered to be complete. However, no diagnoses were recorded for phone, mail or e-mail consultations, and in these cases, the prescriptions could not be linked to a diagnosis.

Antibiotic prescriptions were identified according to Anatomical Therapeutic Chemical Classification (ATC) code group J01, which includes all oral and parenteral antibiotics, but not antibiotics in ointments or eye drops. Antibiotic prescriptions were linked to diagnoses if within a week after a visit. Antibiotic treatment without a diagnosis of an infection could also result from consultation with a care provider other than a physician or from a non-infection diagnosis at a visit. Information on whether the patients collected the medication at the pharmacies was not available in the present study.

### Data set

All physician visits with an infection diagnosis and all antibiotic prescriptions were extracted from the KIDPC database, resulting in a data set with 702,048 physician visits and 389,263 prescriptions over 9 years. For each visit, data on the patient’s age and sex, infection diagnoses, antibiotic treatments, and PHCC were extracted.

A visit was defined as a physical visit to a physician, and a consultation was defined as a phone, mail, e-mail or nurse contact. It was compulsory for the physician to code the diagnosis when documenting the visit. Only physician coded diagnoses were used in this study for consistency. In 3% of the visits more than one infection diagnosis was recorded, and in these cases the main diagnosis was selected based on the severity and the likelihood of the diagnosis resulting in an antibiotic prescription. Consultations were not coded for diagnoses, but could in some instances result in antibiotic prescriptions, for example treatment for UTI or repeat prescriptions.

This study presents descriptive annual data and mean annual change for infections and antibiotic prescribing per 1000 inhabitants divided per main infection group, age group, sex, and per IH and OOH (Tables 1, 2, 3, 4). The data are presented as numbers per 1000 inhabitants per year based on the population of the region as of December 31 of each year. Because the population of Kronoberg County is only 2% of the population of Sweden and the antibiotic prescription rate was lower than the average in Sweden [26], the numbers reported cannot be extrapolated to the national level. However, the trends are likely to be generalisable.

**Table 1:** Visits according to the type of infection per 1000 inhabitants per year

	Visits per 1000 inhabitants and year										
										Average	Mean annual
	2006	2007	2008	2009	2010	2011	2012	2013	2014	2006–2014	change (95% CI)
**All hours**											
Respiratory tract infections^a^	241	258	243	253	255	267	258	234	205	246	− 2.9 (−8.3 to 2.5)
Skin & soft tissue infections	58	59	60	68	67	72	71	71	69	66	1.7 (0.8 to 2.6)
Urinary tract infections	51	52	51	54	55	54	52	51	49	52	−0.2 (−0.7 to 0.4)
Other infections^b^	61	60	61	69	71	76	77	74	75	69	2.2 (1.3 to 3.2)
**Total all hours**	412	430	416	445	448	469	457	430	398	434	0.9 (−6.5 to 8.3)
**In-hours**											
Respiratory tract infections^a^	200	215	207	223	227	238	230	209	182	215	−0.5 (−6.1 to 5.1)
Skin & soft tissue infections	51	52	54	62	62	67	65	65	63	60	1.8 (0.8 to 2.9)
Urinary tract infections	42	43	43	47	48	47	44	45	42	45	0.1 (−0.6 to 0.8)
Other infections^b^	53	53	55	64	66	70	71	68	69	63	2.5 (1.4 to 3.5)
**Total in-hours**	347	364	360	397	402	421	410	387	356	382	3.9 (−4.1 to 11.9)
**Out-of-hours**											
Respiratory tract infections^a^	42	42	36	30	28	30	28	25	23	32	−2.3 (−3.1 to − 1.6)
Skin & soft tissue infections	6.9	7.1	6.0	5.5	5.5	5.9	5.5	5.7	6.4	6.1	−0.1 (−0.3 to 0.1)
Urinary tract infections	9.0	8.9	8.0	7.0	7.3	7.0	7.5	6.6	7.1	7.6	−0.3 (−0.4 to − 0.1)
Other infections^b^	7.7	7.5	6.0	5.4	5.5	5.8	5.7	5.7	5.7	6.1	−0.2 (−0.4 to 0.0)
**Total out-of-hours**	65	66	56	48	46	48	47	43	43	51	−3.0 (−4.2 to −1.7)

^a^ Includes ear infections

^b^ Includes eye infections, gastrointestinal infections, and rare infections

**Table 2:** Visits due to infections according to sex and age group per 1000 inhabitants per year

	Visits per 1000 inhabitants per year										
										Average	Mean annual
	2006	2007	2008	2009	2010	2011	2012	2013	2014	2006–2014	change (95% CI)
**All hours**											
Female	477	499	481	514	517	546	528	500	458	502	0.9 (−8.0 to 9.8)
Male	345	369	351	376	380	393	386	362	336	366	0.5 (−5.9 to 6.8)
Age (years)											
0–4	997	1172	1062	1059	1079	962	958	867	796	995	−33.7 (−56.0 to − 11.5)
5–19	494	498	451	481	492	511	468	433	382	468	−9.7 (− 19.6 to 0.3)
20–39	376	383	353	381	377	406	393	357	331	373	−2.5 (− 9.5 to 4.5)
40–64	319	329	315	348	349	379	368	356	326	343	4.1 (−2.2 to 10.4)
65–79	404	407	396	432	428	471	474	460	431	434	7.7 (1.1 to 14.3)
≥ 80	506	522	552	570	578	610	632	616	595	576	13.9 (7.6 to 20.2)
**In-hours**											
Female	403	425	418	460	464	492	475	451	410	444	4.2 (−5.3 to 13.7)
Male	288	311	303	334	340	351	346	324	299	322	3.1 (−3.7 to 9.9)
Age (years)											
0–4	720	889	851	884	911	816	813	736	666	810	−13.7 (− 38.6 to 11.2)
5–19	375	392	373	412	426	440	403	373	327	391	−2.7 (−13.5 to 8.1)
20–39	296	313	299	335	332	357	345	313	286	320	1.3 (−6.5 to 9.0)
40–64	274	287	282	319	320	347	337	326	297	310	5.9 (−0.8 to 12.5)
65–79	368	371	369	407	405	445	449	436	406	406	9.1 (2.4 to 15.8)
≥ 80	464	482	515	540	551	582	603	589	568	544	16.0 (9.2 to 22.8)
**Out-of-hours**											
Female	74	74	63	54	52	54	53	49	48	58	−3.3 (−4.7 to −1.9)
Male	56	58	49	42	40	42	40	38	37	45	−2.6 (−3.7 to − 1.6)
Age (years)											
0–4	277	283	211	175	168	146	145	130	131	185	−20.0 (− 27.3 to −12.7)
5–19	119	106	78	68	66	71	65	60	55	77	−7.0 (−10.4 to − 3.6)
20–39	80	70	54	46	45	49	48	45	45	53	−3.7 (−6.2 to −1.3)
40–64	45	42	34	29	29	32	31	30	29	33	−1.8 (−3.0 to −0.6)
65–79	36	35	27	25	22	26	25	24	24	27	−1.4 (−2.5 to − 0.4)
≥ 80	42	40	37	30	27	28	29	27	26	32	−2.1 (− 2.9 to −1.2)

**Table 3:** Antibiotic prescriptions according to the type of infection per 1000 inhabitants per year

	Antibiotic prescriptions per 1000 inhabitants per year										
										Average	Mean annual
	2006	2007	2008	2009	2010	2011	2012	2013	2014	2006–2014	change (95% CI)
**All hours**											
Respiratory tract infections^a^	124	135	114	109	111	109	103	85	70	107	−6.5 (−9.0 to − 3.9)
Skin & soft tissue infections	31	32	29	33	30	32	29	30	28	30	−0.3 (−0.7 to 0.1)
Urinary tract infections	44	44	41	44	45	43	40	40	38	42	−0.7 (−1.2 to − 0.1)
Other infections^b^	4.5	4.8	4.3	4.4	4.4	4.9	3.9	3.3	3.4	4.2	−0.1 (−0.3 to 0.0)
Without infection diagnosis^c^	62	63	60	58	57	57	59	55	55	58	−0.9 (−1.4 to − 0.5)
**Total all hours**	266	278	248	249	247	246	236	213	194	242	−8.5 (−11.9 to − 5.2)
**In-hours**											
Respiratory tract infections^a^	101	109	94	93	95	93	88	73	59	89	−4.8 (−7.1 to −2.5)
Skin & soft tissue infections	26	26	25	29	26	28	26	26	24	26	−0.2 (−0.7 to 0.3)
Urinary tract infections	37	36	34	38	38	37	34	34	32	36	−0.5 (−1.1 to 0.1)
Other infections^b^	4.0	4.3	3.9	4.1	4.0	4.4	3.5	3.0	3.2	3.8	−0.1 (−0.2 to 0.0)
Without infection diagnosis^c^	55	57	55	55	54	53	55	51	50	54	−0.7 (−1.1 to − 0.3)
**Total in-hours**	223	233	212	219	217	216	205	186	168	209	−6.3 (−9.6 to −3.0)
**Out-of-hours**											
Respiratory tract infections^a^	23	26	21	16	16	16	16	12	11	17	−1.7 (−2.3 to − 1.1)
Skin & soft tissue infections	4.6	5.1	4.1	3.8	3.8	4.0	3.9	3.9	4.0	4.1	−0.1 (−0.2 to 0.0)
Urinary tract infections	7.3	7.6	6.6	5.9	6.1	5.9	6.5	5.6	6.0	6.4	−0.2 (−0.3 to 0.0)
Other infections^b^	0.4	0.5	0.4	0.3	0.5	0.4	0.4	0.3	0.3	0.4	0.0 (0.0 to 0.0)
Without infection diagnosis^c^	7.2	5.9	4.1	3.6	3.3	3.7	4.2	4.4	4.5	4.5	−0.3 (− 0.6 to 0.1)
**Total out-of-hours**	43	45	36	30	30	30	31	27	26	33	−2.2 (−3.3 to −1.2)

^a^ Includes ear infections

^b^ Includes eye infections, gastrointestinal infections, and rare infections

^c^ Prescriptions with non-infection diagnosis or no diagnosis registered

**Table 4:** Antibiotic prescription according to sex and age group per 1000 inhabitants per year

	Antibiotic prescriptions per 1000 inhabitants per year										
										Average	Mean annual
	2006	2007	2008	2009	2010	2011	2012	2013	2014	2006–2014	change (95% CI)
**All hours**											
Female	249	264	231	237	233	232	218	196	173	226	−9.0 (−13.0 to −5.1)
Male	157	171	147	144	147	146	136	120	105	142	−6.3 (−9.2 to −3.5)
Age (years)											
0–4	472	562	479	440	440	357	347	281	241	402	−35.2 (−46.9 to −23.5)
5–19	240	254	208	200	209	211	197	165	134	202	−11.7 (−17.0 to −6.5)
20–39	185	192	162	163	163	169	155	135	120	160	−7.4 (−10.6 to −4.1)
40–64	168	175	147	158	155	160	146	136	119	152	−5.2 (−8.2 to −2.3)
65–79	213	211	184	199	188	195	188	174	160	190	−5.3 (−8.1 to −2.6)
≥ 80	206	206	205	196	190	188	186	182	174	192	−4.1 (−4.9 to −3.3)
**In-hours**											
Female	207	218	192	205	202	201	186	169	146	192	−6.7 (−10.7 to −2.8)
Male	128	139	122	124	125	125	115	102	89	119	−4.7 (−7.2 to −2.1)
Age (years)											
0–4	330	402	358	347	350	284	271	226	184	306	−22.5 (−33.3 to −11.7)
5–19	178	189	165	164	172	174	158	134	107	160	−7.5 (−12.2 to −2.9)
20–39	141	150	132	138	137	142	128	112	97	131	−5.0 (−8.1 to −1.8)
40–64	141	149	127	141	138	141	128	120	104	132	−3.9 (−6.8 to −1.0)
65–79	192	189	167	184	174	180	172	159	146	174	−4.5 (−7.1 to −1.8)
≥ 80	185	188	187	183	176	174	170	168	161	177	−3.3 (−4.2 to −2.5)
**Out–of-hours**											
Female	43	46	38	32	32	31	32	27	26	34	−2.3 (−3.2 to −1.3)
Male	29	32	25	21	21	21	21	18	16	23	−1.7 (−2.4 to −0.9)
Age (years)											
0–4	142	159	121	92	90	73	75	56	57	96	−12.7 (−16.7 to −8.6)
5–19	62	65	43	36	37	37	38	31	27	42	−4.2 (−6.3 to −2.1)
20–39	44	42	30	25	26	27	27	23	23	30	−2.4 (−3.8 to −1.0)
40–64	27	26	20	17	18	18	17	16	15	19	−1.4 (−2.0 to −0.7)
65–79	21	22	17	15	14	16	16	14	15	17	−0.9 (−1.5 to − 0.3)
≥ 80	21	18	18	13	13	14	16	14	13	16	−0.8 (−1.3 to − 0.2)

The IH and the OOH cohorts were compared. The relative risk of receiving antibiotics during OOH was calculated (Table 5). The proportions of the choice of antibiotics for common infections were reported (Table 6).

**Table 5:** Visits and antibiotic prescriptions per diagnosis for in-hours compared to out-of-hours

Diagnoses	In-hours			Out-of-hours			
	Infection visits	Antibiotic prescriptions		Infection visits	Antibiotic prescriptions		
	Per 1000 inhabitants per year (%)	Per 1000 inhabitants per year	Percent of cases	Per 1000 inhabitants per year (%)	Per 1000 inhabitants per year	Percent of cases	Adjusted relative risk^b^ (95% CI)
**Respiratory tract infections**	215 (56%)	89	42%	32 (62%)	17	55%	1.25 (1.24 to 1.26)
Acute bronchitis	23 (6%)	11	47%	2.2 (4%)	1.0	46%	0.95 (0.91 to 0.98)
Acute otitis media	23 (6%)	20	85%	6.7 (13%)	6.1	91%	1.01 (1.00 to 1.02)
Chronic Obstructive Pulmonary Disease	14 (4%)	2.4	18%	0.4 (1%)	0.1	34%	1.02 (0.87 to 1.19)
Influenza	1.9 (0%)	0.1	6%	0.4 (1%)	0.0	4%	0.75 (0.51 to 1.11)
Pharyngotonsillitis	28 (7%)	23	80%	6.8 (13%)	5.7	84%	1.01 (1.00 to 1.02)
Pneumonia	14 (4%)	9.1	67%	2.2 (4%)	1.7	76%	1.08 (1.05 to 1.10)
Sinusitis	17 (5%)	14	83%	1.8 (4%)	1.6	85%	1.00 (0.98 to 1.02)
Upper respiratory tract infection	81 (21%)	8.0	10%	9.6 (19%)	0.9	9%	0.87 (0.83 to 0.92)
Other respiratory tract infection	12 (3%)	2.2	18%	1.5 (3%)	0.4	27%	1.04 (0.95 to 1.14)
**Skin and soft tissue infections**	60 (16%)	26	44%	6.1 (12%)	4.1	68%	1.20 (1.18 to 1.23)
**Urinary tract infections**	45 (12%)	36	80%	7.6 (15%)	6.4	84%	1.04 (1.04 to 1.05)
Lower urinary tract infections	34 (9%)	31	90%	6.3 (12%)	5.7	89%	1.00 (1.00 to 1.01)
Other urogenital infections	10 (3%)	4.4	44%	1.3 (3%)	0.7	56%	1.07 (1.02 to 1.13)
**Other infections**^**a**^	63 (17%)	3.8	6%	6.1 (12%)	0.4	7%	0.81 (0.74 to 0.89)
**Total**	382 (100%)	155	41%	51 (100%)	28	55%	

^a^ Includes eye infections, gastrointestinal infections, and rare infections

^b^ Relative risk of antibiotic prescription adjusted for sex and age during out-of-hours compared to in-hours

**Table 6:** Antibiotic treatment by antibiotic group for the six most common diagnoses between in-hours and out-of-hours

Indication		Prescription^a^, %	
	Choice of antibiotic	In-hours	Out-of-hours
Acute bronchitis	Doxycycline	59%	52%
(*n* = 18,970)	Phenoxymethylpenicillin	21%	27%
	Amoxicillin	11%	11%
	Macrolides	5%	7%
	Cefadroxil	2%	2%
Acute otitis media	Phenoxymethylpenicillin^b^	70%	69%
(*n* = 41,419)	Amoxicillin	20%	21%
	Macrolides	4%	4%
	Amoxicillin/clavulanate	2%	2%
	Cephalosporins	2%	2%
Lower urinary tract infection	Pivmecillinam^b^	45%	46%
(*n* = 59,335)	Nitrofurantoin^b^	22%	20%
	Quinolones	17%	18%
	Trimethoprim	9%	7%
	Cefadroxil	5%	6%
Pharyngotonsillitis	Phenoxymethylpenicillin^b^	78%	79%
(*n* = 45,547)	Cephalosporins	9%	8%
	Clindamycin	6%	5%
	Macrolides	3%	3%
	Amoxicillin	2%	3%
	Tetracyclines	2%	1%
Pneumonia	Phenoxymethylpenicillin^b^	41%	45%
(*n* = 17,527)	Doxycycline	38%	32%
	Amoxicillin	9%	11%
	Macrolides	8%	7%
	Cefadroxil	2%	2%
Sinusitis	Phenoxymethylpenicillin^b^	54%	60%
(*n* = 23,070)	Tetracyclines	30%	25%
	Amoxicillin	9%	9%
	Macrolides	2%	2%
	Cephalosporins	3%	2%

^a^ Antibiotics with prescribed percentages over 2% are shown

^b^ First-choice antibiotics according to the Swedish prescribing guidelines

### Statistical methods

All analyses were performed using Excel 2013 (Microsoft, Redmond, WA, USA) and SPSS Version 23 (IBM Corp, Armonk, NY, USA). For descriptive statistics, means, and proportions were used. For annual trends, linear regressions were calculated and presented as mean annual change with 95% confidence interval. Comparisons between groups after adjusting for sex, age, and diagnosis were presented as relative risks with 95% confidence interval. Comparisons between proportions of categorical variables in two independent groups were performed with the chi-square test. *P*-values ≤ .05 were considered statistically significant.

## Results

The physician visit rate for infections varied during the study and reached a maximum of 469 visits per 1000 inhabitants per year in 2011 and a minimum of 398 visits in 2014. Female patients have more infection visits than male patients, 502 and 366 visits per 1000 inhabitants per year respectively. Children 0–4 years and adults over 80 years had the highest visit rates, 995 and 576 visits per 1000 inhabitants per year respectively. No significant trends were observed in total visit rate nor in visit rate by sex, but the mean annual change in visit rate per 1000 inhabitants per year decreased in children 0–4 years (− 33.7 (95% CI − 56.0 to − 11.5)), increased in adults 65–79 years (7.7 (95% CI 1.1 to 14.3) and in adults over 80 years (13.9 (95% CI 7.6 to 20.2)) (Tables 1 and 2).

The antibiotic prescriptions per 1000 inhabitants per year decreased significantly from 266 prescriptions in 2006 to 194 prescriptions in 2014 (mean annual change − 8.5 (95% CI − 11.9 to − 5.2)). There was no sex difference, but the decrease in antibiotic prescriptions was more pronounced in children 0–4 years (mean annual change − 35.2 (95% CI − 46.9 to − 23.5)) and in children 5–19 years (mean annual change − 11.7 (95% CI − 17.0 to − 6.5). The antibiotic prescribing frequency decreased mainly for RTIs (mean annual change − 6.5 (95% CI − 9.0 to − 3.9)), explaining 76% of the total reduction. Antibiotic prescriptions without an infection diagnosis and prescriptions for UTIs also decreased, explaining a further 11 and 8% of the total reduction, respectively (Tables 3 and 4).

Of all antibiotic prescriptions, 75% were linked to an infection visit on the same day, another 3% were linked to an infection visit within a week before the prescription day, and finally 22% were not possible to link to an infection visit. These proportions were stable during the study period. Of all antibiotics prescribed at visits, 66% were antibiotics commonly used for RTIs, 12% were commonly used for SSIs, 16% were commonly used for UTIs, and 6% were other antibiotics. Of the antibiotics prescribed without an infection diagnosis, 38% were antibiotics commonly used for RTIs, 25% were commonly used for SSIs, 29% were commonly used for UTIs, and 8% were other antibiotics. Of the UTI antibiotics, 36% were prescribed without an infection diagnosis.

During the study period, the OOH infection visits decreased from 65 visits per 1000 inhabitants in 2006 to 43 visits in 2014 (mean annual change − 3.0 visits (95% CI − 4.2 to − 1.7)). Also, the antibiotic prescribing decreased from 43 prescriptions per 1000 inhabitants in 2006 to 26 prescriptions in 2014 (mean annual change − 2.2 prescriptions (95% CI − 3.3 to − 1.2)).

The diagnoses and antibiotic prescription rates between IH and OOH are shown in Table 5. During IH, there were 382 infection visits per 1000 inhabitants per year compared to 51.4 during OOH. Thus 12% of all visits were during OOH. RTIs were the most common diagnoses during both IH and OOH. However, acute otitis media, pharyngotonsillitis, and lower UTIs were more common during OOH. A total of 15% of all antibiotics were prescribed during OOH. The likelihood of receiving an antibiotic prescription was 55% during OOH visits compared to 41% during IH visits. The unadjusted relative risk of antibiotic prescribing in OOH was 1.37 (95% CI 1.36 to 1.38) compared to IH. The difference remained unchanged when only adjusted for age and sex 1.37 (95% CI 1.37 to 1.38) and 1.37 (95% CI 1.37 to 1.38), respectively. However, when adjusted for age, sex, and diagnosis the relative risk of antibiotic prescribing during OOH was 1.09 (95% CI 1.08 to 1.10) compared to IH. No difference was found between the two OOHCs. Age and sex adjusted relative risks of antibiotic prescribing during OOH per diagnosis were significantly higher for acute otitis media, pharyngotonsillitis, pneumonia, SSI and UTI.

For the six most common diagnoses treated with antibiotics, a comparison of treatment choice per diagnosis with IH and OOH visits was made. The prescription rate was higher during OOH for pneumonia, acute otitis media, and pharyngotonsillitis. Although the difference was statistically significant, the choices of treatment for each diagnosis were comparable between IH and OOH prescriptions (Table 6).

## Discussion

During the study period, the level of infection visits was constant, but the antibiotic prescription rate decreased. Fewer prescriptions in children and for RTIs were the main reasons for the reduction. During OOH, there was a reduction both in infection visits and in antibiotic prescribing. The antibiotic prescription rate was higher during OOH than during IH, and when adjusting for age, sex, and diagnosis the difference was significant but small. The choices of treatments were similar.

This study showed that women visited primary care for infections more often than men and also received antibiotic treatment more often than men. The same pattern has been seen in other studies from Denmark, the Netherlands, and the United Kingdom [10, 27, 28]. The sex difference in the incidence of lower UTI was an important reason.

Our data on visit rates per 1000 inhabitants per years for infections were similar to the Primary Care Record of Infections in Sweden (PRIS) database [15], which consists of data since 2007 on visits with an infectious diagnosis and all antibiotic prescriptions from voluntarily participating PHCCs on an annual basis. Antibiotic prescriptions are in most cases linked to diagnoses and also includes information about age, sex, and laboratory results. The database has a larger dataset than in this study covering PHCCs in other regions but lacks OOH data. In the PRIS database, the visit rates per 1000 persons per year for infections during IH were 457 (in 2008), 441 (in 2010), and 406 (in 2013).

The total antibiotic prescribing in primary care decreased by 27% in this study. However, in the PRIS database [15] the reduction of IH antibiotic prescribing was 36%, as the IH antibiotic prescription per 1000 persons per year decreased from 245 (in 2008) to 201 (in 2010) to 157 (in 2013). For the corresponding years in our study, the IH antibiotic prescriptions per 1000 inhabitants were 212, 217, and 186, respectively. It is possible that participation in the PRIS database could have triggered a more restrictive antibiotic prescribing behaviour compared to our real-life study. A Finnish study [29] reported a 47% reduction in antibiotic prescriptions to children in primary and other out-patient care between 2010 and 2016, whereas our present study showed a 38% reduction in children in primary care between 2010 and 2014.

Several explanations are possible for the reduction in antibiotics prescriptions. For example, there might be increasing awareness among the general public that the use of antibiotics should be avoided when they are not needed. Also, physicians might have become more restrictive in prescribing. Another reason might be due to the antibiotic stewardship work performed by the Strama group, the Swedish strategic programme against antibiotic resistance [30]. In 2005, Strama together with the government launched a national strategy to prevent antibiotic resistance and healthcare-associated infections. Several actions have been performed in relation to this strategy. Diagnosis-specific guidelines for optimal antibiotic use have been published and promoted, and the use of antibiotics has been reported at the local, regional, and national level [17, 31]. During 2011–2014, the Swedish government ran a patient safety campaign aiming to decrease antibiotic use with the goal of fewer than 250 annual prescriptions in out-patient care per 1000 inhabitants for all prescribers together (primary and secondary care, dental care) resulting in a decrease from 385 prescriptions (2011) to 328 prescriptions (2014) [26, 32]. Furthermore, a pneumococcal conjugate vaccine was introduced in the Swedish national vaccination programme for children in 2009. Finally, a national economic bonus system was introduced for regions achieving a reduction in the antibiotic prescription levels, and incentive for quality outcome with the same goal was introduced in 2011 at the PHCC level in Kronoberg County.

During the period studied here, the number of OOH infection visits decreased by a third. Factors contributing to the decrease were shorter opening hours at the end of the study, a penalty fee (100 euros) introduced in 2008 for the PHCC for each patient attending the OOHC, and the introduction of a nurse triage system for walk-in patients at OOHC1.

The OOH antibiotic prescription rate per 1000 inhabitants per year was at the same level in the Netherlands, Sweden, and England (20, 28, and 31 prescriptions, respectively), but higher in Denmark (80 prescriptions) [9, 13, 27]. Two English studies have shown stable or increased OOH antibiotic prescription rates from 2010 to 2014 [8, 9]. In contrast, our study showed a decrease in antibiotic prescription rates.

The main explanation for excess prescribing during OOH is that infections that are often treated with antibiotics were more common during OOH visits such as acute media otitis, pharyngotonsillitis, and lower UTIs. The relative risk of antibiotic prescribing was decreased when adjusting for diagnoses. For SSI, the relative risk of receiving antibiotics during OOH remained elevated 1.20 (95% CI 1.18–1.23). It was uncommon to prescribe UTI antibiotics without a visit with infection diagnosis during OOH service (9% of UTI antibiotic prescriptions were without a visit during OOH compared to 39% during IH) although it was in line with current guidelines. This fully explained the higher UTI visit rate during OOH.

These results are similar to other European studies when comparing OOH and IH. A Norwegian comparison of tonsillitis and acute media otitis showed no difference in the prescription rate at OOHCs [33], and a Dutch study showed higher prescription levels during OOH for common infections and argued that the patients were sicker in the sense that they had more urgent problems that could not wait until the next day based on a revision of the EMR [13].

The remaining excess prescriptions during OOH after adjusting for diagnosis were estimated, leading to 2.2 more prescriptions per 1000 inhabitants per year compared to IH, which corresponds to 7.9% of the prescriptions during OOH and to 1.2% of all prescriptions during IH and OOH together. These prescriptions could partly be explained by sicker patients in need for urgent evaluation and an absence of control visits in the OOH setting. On the other hand, a reason could be a lower threshold to prescribe during OOH for example due to high workload or due to limited possibility to arrange for follow-ups.

Apart from the high relative risk of receiving antibiotics for SSI during OOH, there are no apparent areas to intervene. But because the total decrease of antibiotic prescriptions during the study period is 27% and the excess prescriptions during OOH are just above 1% of all antibiotic prescriptions, there would be limited gain from intervening in the OOH setting.

There were no differences in treatment choice, which corresponds with other quantitative studies from Norway and the Netherlands [13, 33] and with a Belgian qualitative study where physicians reported the treatment choice to be the same as during IH, although the threshold to prescribe was lower at OOHCs [11]. In contrast, an English study noted a higher proportion of broad-spectrum antibiotics during OOH [8].

### Strengths

The data set was complete for infection visits and antibiotic prescriptions in primary care in a region in Sweden. Because the whole region was included, the data were real-life data without any selection due to study participation. Also, the same EMR system was used during the study period thus decreasing the risk for information errors. Because writing a diagnosis was compulsory for all visit records, very few diagnoses were missing. All OOH infection visits and prescriptions were included, which enabled comparisons between IH and OOH, adjusting for sex, age groups, and diagnoses. The comparison between IH and OOH is relevant for Sweden as a whole and for other countries with similar OOH settings.

### Limitations

Limitations of the study include that no validation of diagnoses by examining the EMR was done. Also, the reason why some antibiotics are prescribed without a coded infection diagnosis has not been explored. A lower threshold to diagnose infections and to prescribe antibiotics in the OOH setting cannot be ruled out but would also be hard to verify in the EMR. Other antibiotics than oral and parenteral antibiotics (ATC code J01) are missing in the dataset, such as antibiotics in topical skin and eye preparations. The antibiotic rate for the elderly (> 80 years) might be underestimated due to partly missing data for patients with medication administered through a dispensing system. Furthermore, we could not measure the rate of delayed prescribing because we did not have access to pharmacy dispensing data. The common way of delayed prescribing in Sweden is that the patient receives an electronic prescription but is recommended to wait a few days before collecting the prescription [34].

## Conclusions

Although the infection visit rate was unchanged, there was a significant reduction in antibiotic prescribing, especially to children and for RTIs. The increased antibiotic prescribing rate during OOH was small when adjusted for age, sex, and diagnosis, and no excess prescribing of broad-spectrum antibiotics was seen. Therefore, interventions selectively aiming at OOHCs seem to be unmotivated in a low-prescribing context.

## Supplementary information

**Additional file 1.** Infection diagnoses selected to be included in the Kronoberg Infection Database in Primary care 2006–2014. *Description*: The total number of patients and visits are shown for all providers (physicians, nurses) and for physicians only.

## Data Availability

The datasets used and/or analysed during the current study are available from the corresponding author on reasonable request.

## Abbreviations

ATC: Anatomical therapeutic chemical classification
EMR: Electronic medical records
IH: In-hours
KIDPC: Kronoberg Infection database in primary care
OOH: Out-of-hours
OOHC: Out-of-hours centre
PHCC: Primary healthcare centre
PRIS: Primary care record of infections in Sweden database
RTI: Respiratory tract infection
SSI: Skin and soft tissue infection
UTI: Urinary tract infection
